# Cancer du sein chez l'homme: le dermatologue à quoi dire

**DOI:** 10.11604/pamj.2015.21.63.5952

**Published:** 2015-05-28

**Authors:** Inssaf Ramli, Nadia Ismaili

**Affiliations:** 1 Service de Dermatologie et Vénérologie, CHU Ibn Sina, Université Mohammed V, Rabat, Maroc

**Keywords:** Carcinome mammaire, masculin, ulcération mamelonnaire, breast carcinoma, male, nipple ulceration

## Image en medicine

Le cancer du sein chez l'homme est une affection rare qui représente 0,4 % à 1,2 % de tous les cancers masculins. Il est souvent découvert à un stade tardif malgré son siège superficiel. Les manifestations cutanées semblent être plus fréquentes et plus précoces chez l'homme que chez la femme. Il s'agit le plus souvent d'un nodule cutané fixe et indolore de siège essentiellement rétroaréolaire. Les autres signes cliniques révélateurs sont à type d’écoulement mamelonnaire ou de modifications cutanées du mamelon à type de rétraction, ulcération ou d‘eczématisation. Celles ci peuvent poser un problème de diagnostic différentiel avec la maladie de Paget, la maladie de Bowen, le carcinome basocellulaire ou le mélanome. Tous les types histologiques rencontrés chez la femme sont décrits chez l'homme : le carcinome canalaire infiltrant reste le plus fréquent. Les récepteurs hormonaux à la progestérone et aux oestrogènes sont fréquemment positifs que chez la femme avec des taux souvent élevés. La chirurgie reste la base de traitement. Les inhibiteurs de l'aromatase, hormonothérapie anti-oestrogénique et/ou la polychimiothérapie sont utilisés comme des traitements adjuvants. Le pronostic reste encore réservé étant donné son évolution imprévisible et son potentiel métastatique élevé. Nous rapportons le cas d'un carcinome mammaire gauche chez un homme de 72 ans révélé par une ulcération mamelonnaire gauche. L’écho-mammographie et l’étude histologique étaient en faveur d'un carcinome canalaire infiltrant avec des métastases cutanées. Un bilan d'extension révélait des métastases pulmonaires et osseuses. Le patient est décédé avant de commencer la première cure de polychimoithérapie.

**Figure 1:**
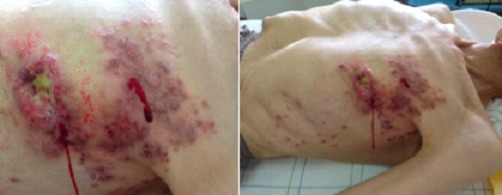
Tumeur mammaire ulcéro- bourgeonnante avec des papules cutanées métastatiques

